# Prediction of patients with a tumor proportion score > 50% who do not respond to first-line monotherapy with pembrolizumab

**DOI:** 10.1186/s12885-020-6582-4

**Published:** 2020-02-03

**Authors:** Mitsunori Morita, Motohiro Tamiya, Daichi Fujimoto, Akihiro Tamiya, Hidekazu Suzuki, Katsuya Hirano, Yasushi Fukuda, Toshihide Yokoyama, Ryota Kominami, Masaki Kanazu, Junji Uchida, Satoshi Hara, Shuji Yamashita, Hiromi Tomioka

**Affiliations:** 1 Department of Respiratory Medicine, Kobe City Medical Center West Hospital, 2-4, Ichiban-cho, Nagata-ku, Kobe-shi, Hyogo 653-0013 Japan; 2 Department of Thoracic Oncology, Osaka International Cancer Institute, 3-1-69 Otemae, Chuo-ku, Osaka, 541-8567 Japan; 3 Department of Respiratory Medicine, Kobe City Medical Center General Hospital, 2-1-1 Minatojimaminamimachi, Chuo-ku, Kobe-shi, Hyogo 650-0047 Japan; 4 Department of Internal Medicine, National Hospital Organization Kinki-Chuo Chest Medical Center, 1180, Nagasone-cho, Kita-ku, Sakai-shi, Osaka, 591-8555 Japan; 5 Department of Thoracic Oncology, Osaka Habikino Medical Center, 3-7-1, Habikino, Habikino-shi, Osaka, 583-8588 Japan; 6 Department of Respiratory Medicine, Hyogo Prefectural Amagasaki General Medical Center, 2-17-77, Higashi-Naniwa-Cho, Amagasaki-shi, Hyogo 660-8550 Japan; 7 Department of Respiratory Medicine, Kurashiki Central Hospital, 1-1-1, Miwa, Kurashiki-shi, Okayama, 710-8602 Japan; 8 Department of Respiratory Medicine, National Hospital Organization Himeji Medical Center, 68, Honmachi, Himeji-shi, Hyogo 670-8520 Japan; 9 Department of Thoracic Oncology, National Hospital Organization Osaka Toneyama Medical Center, 5-1-1, Toneyama, Toyonaka-shi, Osaka, 560-0045 Japan; 10 Department of Respiratory Medicine, Osaka General Medical Center, 3-1-56, Bandai-Higashi, Sumiyoshi-ku, Osaka, 558-8558 Japan; 11 Department of Respiratory Medicine, Itami City Hospital, 1-100, Koyaike, Itami-shi, Hyogo 664-8540 Japan

**Keywords:** Non-small cell lung cancer, Pembrolizumab, First-line therapy, Efficacy, Programmed death ligand-1

## Abstract

**Background:**

Pembrolizumab is effective as first-line therapy against advanced non-small cell lung cancer (NSCLC) in patients with programmed death ligand-1 (PD-L1) expression levels ≥50% [1]. However, it is not effective in all patients, and the factors predicting responses among this population remain unknown.

**Methods:**

We retrospectively analyzed patients with NSCLC and a PD-L1 tumor proportion score (TPS) > 50%, who received first-line monotherapy with pembrolizumab from February 1, 2017 to April 30, 2018. The study included 11 hospitals, which participated in the Hanshin Oncology clinical Problem Evaluation group (HOPE). We analyzed the differences between responders and non-responders in terms of age, sex, performance status score, degree of progression, histological type, smoking history, expression of PD-L1, use of steroids prior to treatment, metastasis site, and laboratory data.

**Results:**

A total of 205 patients were included in this study. Of those, 108 patients exhibiting complete or partial response were defined as responders. Those exhibiting progressive disease (*N* = 52) were defined as non-responders. In the univariate analysis, Eastern Cooperative Oncology Group performance status score ≥ 2 (*p* = 0.0832), stage IV disease or recurrence (*p* = 0.0487), PD-L1 TPS 50–89% (*p* = 0.0657), use of steroids prior to the administration of pembrolizumab (*p* = 0.0243), malignant pleural effusion (*p* = 0.0032), and baseline C-reactive protein (CRP) levels > 1.0 mg/dL (*p* = 0.0390) were significantly associated with non-response to treatment.

In the multivariate analysis, use of steroids prior to the administration of pembrolizumab (odds ratio [OR]: 5.86; 95% confidence interval [CI]: 1.32–31.8; *p* = 0.0200), malignant pleural effusion (OR: 2.68; 95% CI: 1.15–6.35; *p* = 0.0228), and baseline CRP > 1.0 mg/dL (OR: 2.17; 95% CI: 1.03–4.68; *p* = 0.0402) were significantly associated with non-response to treatment.

**Conclusion:**

In real-world patients with NSCLC and a PD-L1 TPS ≥50%, use of steroids prior to treatment, malignant pleural effusion, and baseline CRP levels > 1.0 mg/dL reduced the response of first-line monotherapy with pembrolizumab.

## Background

Non-small-cell lung cancer (NSCLC) is the leading cause of cancer-related death worldwide, and the outcomes for patients with NSCLC are currently poor [2] . However, the development of programmed cell death (PD)-1 immune checkpoint inhibitors (ICIs) has improved treatment outcomes for NSCLC [3–5]. The results of the international, randomized, open-label, phase 3 KEYNOTE-024 trial showed that, in patients with advanced NSCLC and programmed cell death ligand-1 (PD-L1) expression levels ≥50% in tumor cells, treatment with pembrolizumab was associated with significantly longer progression-free survival (PFS) and overall survival (OS), and fewer adverse events versus platinum-based chemotherapy [1, 6].

Pembrolizumab has dramatically changed the first-line standard therapy for patients with high levels (≥50%) of PD-L1 expression in daily clinical practice. Furthermore, in the KEYNOTE-024 trial, the objective response rate in the pembrolizumab group was 44.8%. Numerous cases have demonstrated the significant therapeutic effect of pembrolizumab in the real-world setting. However, in a number of cases, treatment with pembrolizumab is ineffective. Using real-world data obtained from multiple institutions (including city hospitals), in the present study, we examined patients who did not respond to monotherapy with pembrolizumab despite exhibiting high levels of PD-L1 expression.

## Methods

This was a multicenter, observational, retrospective cohort study involving patients with NSCLC who received first-line monotherapy with pembrolizumab.

We investigated a total of 213 patients from 11 hospitals participating in the Hanshin Oncology clinical Problem Evaluation group (HOPE) from February 1, 2017 to April 30, 2018.

The effect of the treatment was evaluated according to the Response Evaluation Criteria in Solid Tumors: Revised (RECIST) guideline (version 1.1). Eight patients were excluded for the following reasons: changing hospital (*N* = 3), death prior to the evaluation of the treatment effect (*N* = 2), drug-induced lung injury and switch to a subsequent treatment (N = 2), recurrence of brain metastasis, and use of radiotherapy (*N* = 1). Therefore, a total of 205 patients were included in the analysis. Patients were followed-up for disease status until August 31, 2018.

### Statistical analysis

For all statistical analyses, the JMP® statistical software program (12th version; SAS Institute Inc., Cary, NC, USA) was used. Categorical variables were analyzed using the χ2 test or Fisher’s exact test. A *p* < 0.05 denoted statistically significant differences. Multivariate logistic regression analysis was performed by selecting factors with *p* < 0.10 in the univariate analysis. The Kaplan–Meier method was used to produce the survival curve.

This study was approved by the institutional review board of each participating hospital, and is registered with the UMIN (University Hospital Medical Information Network in Japan; number 000032470).

## Results

### Patient characteristics

The clinical characteristics of the 205 patients are shown in Table 1. The median age at the time of treatment with pembrolizumab was 70 years (range: 44–91 years). The majority of the patients (*N* = 170; 82.9%) were males, and 169 patients (82.4%) had an Eastern Cooperative Oncology Group performance status (ECOG PS) of 0–1. Most patients (*N* = 168; 82.0%) had stage IV disease or recurrence; however, a number of patients with stage II and III disease were inoperative and unable to receive radiation therapy. The tissue types were squamous cell carcinoma (*N* = 54; 26.3%), adenocarcinoma (*N* = 123; 60.0%), NSCLC not otherwise specified (*N* = 22; 10.7%), pleomorphic carcinoma (*N* = 4; 2.0%), spindle cell carcinoma (*N* = 1; 0.5%), and large cell carcinoma (N = 1; 0.5%). Six patients were epidermal growth factor receptor mutation-positive: exon 19 deletion (N = 2); exon 19 deletion+T790 M (N = 1); G719A (N = 2); and G719C (N = 1). A total of 182 patients (88.8%) had smoking history. There were 138 patients (67.3%) with a tumor proportion score (TPS) of 50–90%, and 67 patients (32.7%) with a TPS of 90–100%. Of note, there were 13 patients (6.3%) who received steroids prior to the initiation of treatment with pembrolizumab (Table 1).

**Table 1:** Patient characteristics at baseline

Characteristics	Patients (*N* = 205)
Median age (range), years	70 (44–91)
Gender: male / female	170 / 35
ECOG PS score: 0 / 1 / 2 / 3 / 4	49 / 120 / 29 / 6 / 1
Stage: II / III / IV recurrence	3 / 34 / 130 / 38
Histological types: ADC / SCC / NSCLC-NOS / other	123 / 54 / 22 / 6^a^
EGFR mutation: positive / negative / unknown	6^b^ / 171 / 28
Smoking history: ever / never / unknown	182 / 19 / 4
PD-L1: 50–89% / 90–100%	138 / 67
Steroid use: yes / no	13 / 192
Best response: CR / PR / SD / PD	3 / 105 / 45 / 52

*Abbreviations*: *ECOG PS*, Eastern Cooperative Oncology Group performance status, *ADC* adenocarcinoma, *SCC* squamous cell carcinoma; *NSCLC-NOS* non-small cell carcinoma -not otherwise specified, *EGFR*, epidermal growth factor receptor, *PD-L1* programmed cell death ligand 1, *CR* complete response, *PR* partial response, *SD* stable disease, *PD* progressive disease, *Ex19del* exon 19 deletion

^a^pleomorphic carcinoma: four cases; spindle cell carcinoma: one case; large cell carcinoma: one case

^b^Ex19del: two cases; Ex19del + T790 M: one case; G719A: two cases; G719C: one case

### Difference in treatment effectiveness

Complete response (CR), partial response (PR), stable disease (SD), or progressive disease (PD) was observed in three, 105, 45, and 52 patients, respectively. The response rate was 52.7% and the disease control rate was 74.6%.

In this study, we classified the patients into two groups: responders (108 patients exhibiting CR or PR) and non-responders (52 patients exhibiting PD).

We compared the baseline characteristics of responders and non-responders in terms of age, sex, performance status score, degree of progression, histological type, smoking history, expression of PD-L1, use of steroids prior to treatment, metastasis site, and laboratory data.

In the univariate analysis, ECOG PS score ≥ 2 (*p* = 0.0832), stage IV disease or recurrence (*p* = 0.0487), PD-L1 TPS 50–89% (*p* = 0.0657), use of steroids prior to the administration of pembrolizumab (*p* = 0.0243), malignant pleural effusion (*p* = 0.0032), and baseline C-reactive protein (CRP) levels > 1.0 mg/dL (*p* = 0.0390) were significantly associated with non-response to treatment (Table 2).

**Table 2:** Univariate analysis

Factor	Responder	Non-responder	*p* value
	(*N* = 108)	(*N* = 52)	
Age > 70 years	59 (54.6)	29 (55.8)	0.8920
Female sex	16 (14.8)	10 (19.2)	0.4782
ECOG PS score ≥ 2	15 (13.9)	13 (25.0)	0.0832
Stage: IV, recurrence	81 (75.0)	46 (88.5)	0.0487
Squamous cell carcinoma	29 (26.9)	15 (28.9)	0.7913
Never smoker	9 (8.3)	5 (9.6)	0.7881
PD-L1 50–89%	65 (60.2)	39 (75.0)	0.0657
Steroid use	3 (2.8)	6 (11.5)	0.0243
Metastasis Brain	14 (13.0)	12 (23.1)	0.1043
Liver	12 (11.1)	8 (15.4)	0.4439
Bone	28 (25.9)	14 (26.9)	0.8932
Adrenal	17 (15.7)	10 (19.2)	0.5809
Pulmonary	31 (28.7)	20 (38.5)	0.2148
Pleural effusion	17 (15.7)	19 (36.5)	0.0032
Laboratory data			
Neutrophil-to-lymphocyte ratio (< 3)	30 (27.8)	14 (26.9)	0.9097
C-reactive protein (< 1.0 mg/dL)	54 (50.0)	17 (32.7)	0.0390
Lactate dehydrogenase (< 240 IU/L)	79 (73.2)	33 (63.5)	0.2105
Albumin (< 3.5 g/dL)	47 (43.5)	26 (50.0)	0.4407

*ECOG PS* Eastern Cooperative Oncology Group performance status, *PD-L1* programmed cell death ligand 1

ECOG PS score ≥ 2, stage IV disease or recurrence, a TPS of 50–90%, use of steroids prior to treatment, the presence of pleural effusion, and baseline CRP levels > 1.0 mg/dL yielded a *p* < 0.10 in the univariate analysis, and were included in the multivariate analysis.

In the multivariate analysis, use of steroids prior to the administration of pembrolizumab (odds ratio [OR]: 5.86; 95% confidence interval [CI]: 1.32–31.8; *p* = 0.0200), the presence of malignant pleural effusion (OR: 2.68; 95% CI: 1.15–6.35; *p* = 0.0228), and baseline CRP levels > 1.0 mg/dL (OR: 2.17; 95% CI: 1.03–4.68; *p* = 0.0402) were significantly associated with non-response to treatment (Table 3).

**Table 3:** Multivariate logistic regression analysis

Factor	Odds ratio	95% confidence interval	*p* value
ECOG PS score ≥ 2	1.44	0.57–3.59	0.4366
Stage: IV, recurrence	1.65	0.61–5.02	0.3357
PD-L1 50–89%	1.91	0.88–4.30	0.1011
Steroid use	5.86	1.32–31.8	0.0200
Metastasis pleural effusion	2.68	1.15–6.35	0.0228
C-reaction protein (< 1.0 mg/dl)	2.17	1.03–4.68	0.0402

*ECOG PS* Eastern Cooperative Oncology Group performance status, *PD-L1* programmed cell death ligand 1

We further analyzed 52 patients (non-responders) who presented PD after monotherapy with pembrolizumab. After the administration of pembrolizumab, the ECOG PS score decreased in 25 patients (48.1%). Second-line treatment was administered in 35 patients (67.3%); however, best supportive care was applied in 17 patients (32.7%).

**Fig. 1:**
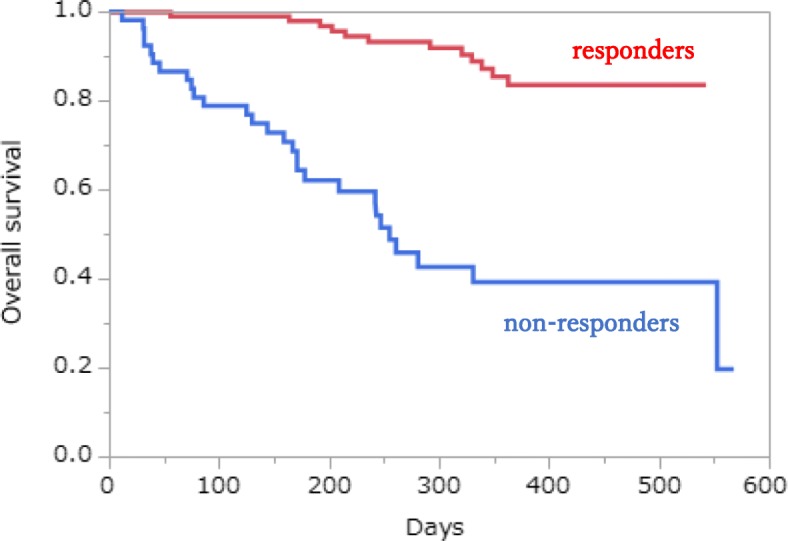
Overall survival in responders and non-responders who received pembrolizumab as first-line therapy

Among those who received second-line treatment, 19 patients achieved PR, seven patients exhibited stable disease, and nine patients experienced PD. The median OS in non-responders was 255 days with poor prognosis. (Fig. 1).

## Discussion

Pembrolizumab has been shown to be effective as primary treatment in NSCLC patients with PD-L1 expression levels ≥50%. However, it is not necessarily effective in all patients. Therefore, the prediction of non-response is of crucial importance in determining the most appropriate treatment regimen.

Based on the results of this retrospective cohort study, pleural effusion, baseline CRP levels > 1.0 mg/dL, and use of steroids prior to treatment tended to reduce the effectiveness of first-line monotherapy with pembrolizumab.

Firstly, we investigated the association between the use of steroids and the effectiveness of pembrolizumab. Taniguchi et al. reported that, in patients treated with nivolumab, ECOG PS score ≥ 2, use of steroids at baseline, and lactate dehydrogenase levels > 240 IU/L were significantly associated with poor PFS [7]. Arbor et al. reported that use of corticosteroids (≥10 mg prednisone or equivalent) at baseline was associated with poorer outcome in patients with NSCLC, who were treated with PD-(L)1 blockade [8]. These studies included patients with any PD-L1 status and lines of therapy.

This study investigated only treatment-naive patients with high expression levels of PD-L1. Consistent with previous reports, treatment with the ICI tended to be less effective in patients who had received prior treatment with steroids.

Secondly, we investigated the association between CRP and response to ICI. Oya et al. reported that, among patients treated with nivolumab, the objective response rate in those with elevated CRP levels (≥1.0 mg/dL) was significantly worse than that reported in patients without elevated CRP levels (< 1.0 mg/dL) [9]. In addition, Inoue et al. reported that, among patients treated with nivolumab, a CRP-to-albumin ratio > 0.3 was associated with early death mainly due to PD and/or the occurrence of immune-related adverse events [10]. Although these are reports of nivolumab, in the present study, pembrolizumab (another PD-1 inhibitor) demonstrated similar findings.

Thirdly, to the best of our knowledge, few studies have assessed the therapeutic effects of ICIs in patients complicated with pleural effusion. Kang et al. reported the response rate in advanced NSCLC patients treated with PD1/PD-L1 inhibitors. The results showed that the response rate was markedly lower in patients with pleural or pericardial metastasis than that observed in those without pleural or pericardial metastasis [11]. Shibaki et al. reported that the presence of malignant pleural effusion was an independent negative predictor affecting PFS and OS, regardless of the presence of positive PD-L1 expression [12]. Furthermore, these studies showed that ICI monotherapy tended to be less effective in patients with pleural effusion. However, the mechanism responsible for the low efficacy of ICIs observed in patients with pleural effusion remains to be elucidated [11, 12].

The KEYNOTE-189 study investigated patients with previously untreated metastatic non-squamous NSCLC without epidermal growth factor receptor or anaplastic lymphoma kinase mutations. In that study, the addition of pembrolizumab to standard chemotherapy (i.e., pemetrexed and a platinum-based drug) resulted in significantly longer OS and PFS versus chemotherapy alone [13]. Moreover, the KEYNOTE-407 study examined patients with previously untreated metastatic, squamous NSCLC. In that study, the addition of pembrolizumab to chemotherapy (i.e., carboplatin plus paclitaxel or nab-paclitaxel) resulted in significantly longer OS and PFS versus chemotherapy alone [14].

In daily practice, it may be difficult to decide whether to choose pembrolizumab monotherapy or combination therapy (i.e., platinum-based chemotherapy and ICI), especially for patients with high PD-L1 expression. Our study may be helpful in selecting treatments among many treatment options. Further studies investigating the use of ICI monotherapy or combination therapy from an efficacy, safety, and medical cost perspective are warranted.

The present study was characterized by several limitations. Firstly, this was a retrospective study. Secondly, treatment effectiveness was evaluated based on the routine practice of each physician. Thirdly, examination of pleural effusion was not performed in all patients. The presence of malignant pleural effusion was defined as not only cytology but also clinical diagnosis such as clinical course and imaging findings. Fourthly, we have not considered details about previous steroid use such as the reason, duration, relation with lung cancer and when the patients received steroid.

## Conclusions

Our study may help to predict patients in whom first-line monotherapy with pembrolizumab is not effective despite the presence of a PD-L1 TPS ≥50%. In particular, the use of steroids prior to the administration of pembrolizumab, the presence of malignant pleural effusion, and baseline CRP levels > 1.0 mg/dL tended to reduce the response of ICI monotherapy.

## Additional file

**Additional file 1.** Institutional Review Board in Osaka International Cancer Institute (approval No.1802199367) and each institution.

## Data Availability

The datasets generated during and/or analyzed during the current study are available from the corresponding author on reasonable request.

## Abbreviations

CR: Complete response
CRP: C-reactive protein
ECOG PS: Eastern Cooperative Oncology Group performance status
ICI: Immune checkpoint inhibitor
NSCLC: Non-small cell lung cancer
OS: Overall survival
PD: Progressive disease
PD-L1: Programmed death ligand-1
PFS: Progression-free survival
PR: Partial response
RECIST: Response Evaluation Criteria in Solid Tumors
SD: Stable disease
TPS: Tumor proportion score
