# Supplementary material for: Prediction of patients with a tumor proportion score > 50% who do not respond to first-line monotherapy with pembrolizumab
Source: BMC Cancer. 2020 Feb 3;20:93. doi: 10.1186/s12885-020-6582-4 (PMC6998183; doi:10.1186/s12885-020-6582-4)
Supplement: Supplementary file 1 — Additional file 1. Institutional Review Board in Osaka International Cancer Institute (approval No.1802199367) and each institution. [file 12885_2020_6582_MOESM1_ESM.docx]

**supplementary file 1**

'Ethics Approval and consent to participate

'

This retrospective study was approved by the Institutional Review Board in Osaka International Cancer Institute (approval No.1802199367), the Clinical Research Review Committee in Kobe City Medical Center West Hospital (approval No.17-019), the Research Ethics Review Committee in Kobe City Medical Center General Hospital (approval No. zn180402), the Clinical Research Review Committee in National Hospital Organization Kinki-Chuo Chest Medical Center (approval No.631), the Medical Research Ethics Committee in Osaka Habikino Medical Center (approval No.892), the Ethics Committee in Hyogo Prefectural Amagasaki General Medical Center (approval No.29-161), the Medical ethics committee in Kurashiki Central Hospital (approval No.2825), the Clinical Research Review Committee in National Hospital Organization Himeji Medical Center (approval No.29-41), the Clinical Research Review Committee in National Hospital Organization Osaka Toneyama Medical Center (approval No. TNH-20180015), the Clinical Research Review Committee in Osaka General Medical Center (approval No. 30-S03-005), and the Research Ethics Committee in Itami City Hospital (approval No.295-1).
